# Haemostatic spray in the management of acute nonvariceal upper gastrointestinal bleeding in children: A single‐centre experience in Singapore

**DOI:** 10.1002/jpr3.12105

**Published:** 2024-07-02

**Authors:** Christopher Wen Wei Ho, Lynette Suk‐hui Goh, Lay Queen Ng, Charanya Rajan, Veena Logarajah, Fang Kuan Chiou

**Affiliations:** ^1^ Gastroenterology, Hepatology and Nutrition Service, Department of Paediatrics KK Women's and Children's Hospital Singapore

**Keywords:** endoscopy, GI bleeding, haemostasis, paediatrics

## Abstract

**Introduction/Objectives:**

Haemostatic spray (HS; Hemospray) is a powder agent for endoscopic haemostasis in patients with acute upper gastrointestinal bleeding (UGIB). It has been shown to be effective and easy to administer. However, published data on efficacy and safety in children remain scarce. Our aim was to describe our experience with the use of HS in the management of UGIB.

**Patients and Methods:**

A retrospective review was conducted of patients aged 0–18 receiving HS for endoscopic haemostasis from January 2017 to December 2021. Information was obtained on demographics, clinical presentation and comorbidities. Outcomes were successful initial haemostasis and rates of re‐bleeding.

**Results:**

A total of 25 applications of HS occurred in 23 patients. The median patient age was 8 years (range: 4 months to 16 years). HS was used in 17/25 (68%) applications as monotherapy. Other treatments employed were clip application and adrenaline injection. One hundred per cent initial haemostasis was achieved with three (13.0%) patients who experienced re‐bleeding. All patients tolerated HS applications with no adverse events.

**Conclusions:**

Our finding supports the use of HS in the management of UGIB in children. HS, either as monotherapy or in combination with other conventional therapy, could potentially be the treatment of choice in children with UGIB with its excellent feasibility and good safety profile.

## Abstract (highlights)

**What is Known:**

- Haemostatic spray (HS) has been shown to be effective and easy to administer for endoscopic haemostasis in patients with acute upper gastrointestinal bleeding (UGIB).
- It has the potential to improve outcomes of acute UGIB in children.

**What is New:**

- HS was highly efficacious in the treatment of paediatric acute UGIB, either as monotherapy or in combination with other conventional therapies.
- 100% initial haemostasis was achieved with a 13% re‐bleeding rate.
- No adverse events were encountered.

## 1 INTRODUCTION

Acute upper gastrointestinal bleeding (UGIB) is an uncommon, but potentially life‐threatening condition in childhood. Mortality associated with gastrointestinal (GI) bleed in children was reported to range from 2% to 15%.^1^, ^2^ This wide range is related to the diversity of populations, the cause of the bleed, which differs according to age, and the competence of the endoscopist.^3^

Endoscopy is the intervention of choice as it is both diagnostic and therapeutic, particularly the application of life‐saving endoscopic therapy. Conventional endoscopic treatment options for GI bleeding include injection of adrenaline, mechanical devices (endoscopic clips, bands) and thermocoagulation techniques (monopolar and bipolar coagulation, argon plasma coagulation and laser photocoagulation).^4^ Advances in endoscopic therapeutic techniques have emerged in the management of UGIB in adults in the last few decades, whereas for the paediatric population, capability in these newer techniques remains very variable.^5^

Topical haemostatic powders have emerged as a valuable tool, due to its ease of use, applicability across various bleeding pathologies and its potential to treat large areas in which the exact location of bleeding is unknown. Hemospray (Cook Medical), initially known as TC‐325, is a mineral‐based haemostatic powder designed to achieve haemostasis by combining the following processes: creation of a physical barrier, absorption of serum fluid components for concentration of clotting factors, and activation of the clotting cascade.^6^, ^7^ It is delivered through a catheter via the working channel of an endoscope with carbon dioxide propellant. It is non‐toxic, neither absorbed nor metabolised by the body, and is eliminated from the stomach and duodenum within 72 h of administration. Hemospray has been approved by the Food and Drug Administration for non‐variceal UGIB and has a CE mark (European approval) for its use in children.^4^

Studies performed mostly in adult patients have reported successful haemostasis rate and low re‐bleeding risk with the use of haemostatic spray (HS).^8^ Published data on application of HS in children remain limited to small cohorts and case reports at present. The aim of this clinical audit is to report the efficacy and safety of using an HS in a retrospective cohort of children who presented with UGIB at a single tertiary paediatric centre in Singapore.

## 2 METHODS

This was a retrospective clinical audit of all paediatric patients aged 0–18 years treated with HS for endoscopic haemostasis of non‐variceal UGIB from January 2017 to December 2021 at KK Women's and Children's Hospital (KKH), which represents the largest paediatric referral centre in Singapore. The requirement for formal institutional board review and approval was waived for the purpose of this audit.

Information was obtained on demographics, family history and clinical presentation, including cardiovascular status, haematemesis, presence of melaena, need for blood transfusion, drop in haemoglobin and comorbidities.

Procedures were performed by two trained paediatric endoscopists. The outcomes collected were initial haemostasis and re‐bleeding. Initial haemostasis was defined as adequate haemostasis confirmed on observation and to the satisfaction of endoscopist. Re‐bleeding was defined as clinical presentation of haematemesis or melaena, haemodynamic instability or a drop in haemoglobin 2 g/L. Re‐bleeding was classified as early (within 72 h) or late (between 72 h and 28 days). Adverse events and equipment failure relating to the use of HS were reported.

Standard treatments employed included the following: adrenaline injection and endoclip application. HS was delivered through a 7 F catheter (Cook Medical) fed through the working channel of at least 2.8 mm size of a standard endoscope (Olympus).

All patients received treatment according to institutional standard of care, including medications (e.g., proton pump inhibitor) as needed. Follow‐up endoscopy was performed at the discretion of the physician to visually confirm an active early or late recurrent bleeding episode.

## 3 RESULTS

A total of 25 applications of HS occurred in 23 patients. The median patient age was 8 years (range: 4 months to 16 years) and 13 patients were boys. Nine cases had significant past medical history, including three with GI disorders, three with haematological diseases, one with metabolic disorder, one with neurological disease and one with cardiac disease. Nine cases had significant past medical history, including three with GI disorders, three with haematological diseases, one with metabolic disorder, one with neurological disease and one with cardiac disease. These included conditions like trachea‐oesophageal fistula, epilepsy and hypothyroidism, and haematological diseases such as leukaemia and aplastic anaemia.

Twenty‐one out of 23 patients presented with haematemesis, 2 patients had the presence of melaena only and 7 had haemodynamic instability on presentation. Mean haemoglobin at presentation was 10.82 ± 2.83 g/dL and five patients had a haemoglobin drop of more than 2 g/dL.

Five patients received fluid bolus for resuscitation and 19 patients had need for blood product transfusion. All patients received intravenous proton pump inhibitor and enteral sucralfate before endoscopy, as part of institutional practice. The dosages used were intravenous omeprazole 1 mg/kg/dose three times a day or intravenous esomeprazole 0.1 mg/kg/h continuous infusion. Four patients received octreotide at 3 µg/kg/h, in view of severity of bleed. Sources of UGIB in the cohort were: gastric ulcer (*n* = 9), erosive pangastritis (*n* = 10), duodenal ulcer (*n* = 3) and oesophageal ulcer (*n* = 1). Aetiologies seen included infective (*Helicobacter pylori* [*n* = 2], fungal [*Candida tropicalis*] [*n* = 1], adenovirus [*n* = 1]), eosinophilic (*n* = 5) and Henoch–Schonlein purpura (*n* = 1). All patients had initial haemostasis, visually confirmed, to the satisfaction of endoscopist after the procedure, with 3 out of 23 (13.0%) patients experiencing re‐bleeding, 2 with early re‐bleeding within 72 h and 1 with late re‐bleeding.

HS was used in 16 of the 23 (70%, 17 applications) patients as monotherapy, with 9 patients having erosive pangastritis and 6 with peptic ulcers (4 gastric ulcers, 2 duodenal ulcers). Re‐bleeding was seen in one patient. This is a child with myelodysplastic syndrome and haematopoietic stem cell transplantation. She did not receive additional therapy due to severity of comorbidities. Table 1 summarises the demographics, clinical and laboratory features of our cohort.

**Table 1:** Demographics, clinical features and laboratory parameters of patients treated with HS.

Demographics and clinical features	Number of patients (*N* = 23)
Age, median (years)	8 (4 months to 16 years)
Gender	13 males (56.5%)
Ethnicity	
Chinese	12
Indian	2
Malay	5
Others	4
Significant pre‐existing condition	9 (39.1%)
Gastrointestinal pathology	3
Metabolic	1
Neurologic	1
Haematological	3
Cardiovascular	1
Presence of haematemesis	21 (91.3%)
Presence of melaena only	2 (8.7%)
Haemodynamic instability (tachycardia or hypotension)	7 (30.4%)
Pathologies leading to UGIB	
Gastric ulcer	9 (39.1%)
Fungal	1
Eosinophilic	1
*Helicobacter pylori*	1
Adenovirus	1
Stress ulcer	1
Idiopathic	4
Erosive pangastritis	10 (43.5%)
Eosinophilic	4
Henoch–Schonlein purpura	1
Stress gastritis	1
Idiopathic	4
Duodenal ulcer	3 (13.0%)
*Helicobacter pylori*	1
Idiopathic	2
Oesophageal ulcer	1 (4.3%)
Initial haemoglobin (g/dL)	10.82 (7.8–14.1)
Haemoglobin drop of >2 g/dL	5 (21.7%)
Platelet count (×10^9^/L)	260 (12–505)
Activated partial prothrombin time (s)	14.49 (12.6–19.4)
Prothrombin time (s)	40.29 (23.7–66.1)
Need for fluid bolus	5 (21.7%)
Need for blood transfusion	14 (60.9%)
Need for other blood products	5 (21.7%)
Ulcer	*N* = 12
Diameter	
<1 cm	11
1–2 cm	1
Location	
stomach	9
Duodenum	3
Forrest classification 1b	12
Re‐bleeding rate	3 (13.0%)
Early	2
Late	1

*Note*: Categorical variables expressed as number (percentage), continuous variables expressed as mean (range).

Abbreviations: HS, haemostatic spray; UGIB, upper gastrointestinal bleeding.

The three patients with re‐bleeding had gastric ulcers, one with fungal gastropathy and underlying acute lymphoblastic leukaemia, one with underlying pancytopaenia, and one with myelodysplastic syndrome and haematopoietic stem cell transplantation. All three had significant thrombocytopaenia, ranging from 12 × 10^9^/L to 74 × 10^9^/L. Table 2 summarises the characteristics of the patients with re‐bleeding.

**Table 2:** Characteristics of patients with re‐bleeding.

Case	Comorbidity	Pathology	Intervention	Initial haemoglobin (g/dL)	Platelet (×10^9^/L)	Prothrombin time (s)	Activated partial prothrombin time (s)
1	Acute lymphoblastic leukaemia	Gastric ulcers, Fungal gastropathy	HS, adrenaline injection	10.5	42	14.1	37.7
2	Cerebral palsy, epilepsy, portal hypertension	Gastric ulcers	HS, adrenaline injection, clip	11.7	74	14.4	39.3
3	Myelodysplastic syndrome, haematopoietic stem cell transplantation	Gastric ulcer, small bowel bleed	HS	8.4	12	17.2	40.2

Abbreviation: HS, haemostatic spray.

Out of three cases, one received monotherapy and two had re‐bleeding despite the use of other standard endohaemostatic techniques. Two patients underwent successful haemostasis by reapplication of HS with other standard techniques. None of the cases required further surgical or non‐endoscopic interventions. One patient did not receive additional therapy due to the severity of comorbidities and medical futility.

All patients tolerated HS applications with no adverse events. Equipment failures reported included blockage through the application catheter, failure of trigger mechanism, breakage or malfunction of catheter or cartridge. Special care also had to be taken to dry the working channel before application of HS. In our cohort, there was no HS‐related equipment failure.

## 4 DISCUSSION

We report our experience with HS for treatment of UGIB in paediatrics. Our audit has demonstrated that HS, either as monotherapy or in combination with other conventional therapy, was highly efficacious in the treatment of paediatric UGIB with a good safety profile. Our findings add to the growing literature on the efficacy and safety of HS use in the paediatric population.

A meta‐analysis of 11 prospective adult studies reported acute haemostasis of 93% in UGIB with 14.4% risk of re‐bleeding episodes.^8^ Limited data is available on the use of HS in children. A prospective study on the use of HS in children (range: 2 days to 17.75 years), compared with conventional endo‐haemostatic techniques, showed that both approaches were comparable in terms of safety and efficacy.^9^ Both groups had achieved initial haemostasis in 100%, with 18% re‐bleeding in the HS group. compared to 24% in the conventional group. These outcomes were similar to what we have experienced in our series. Although the number was small, all cases of re‐bleeding in our cohort had an underlying haematologic disorder with significant thrombocytopaenia which could be the key risk factor leading to re‐occurrence of UGIB. In our experience, the re‐application of HS in conjunction with other conventional techniques was eventually definitive in achieving haemostasis with no further re‐bleeds.

Other published data on the use of HS in paediatric patients are mostly case reports, including an 11‐month‐old infant with a large actively bleeding post‐sclerotherapy oesophageal ulcer and a 2‐year‐old girl with acute liver failure and primary biliary cirrhosis with portal hypertension and bleeding after sclerotherapy, both treated successfully with HS.^10^, ^11^

Feasibility of HS use in younger children may be limited by the size of endoscope used, as a paediatric gastroscope will not allow therapeutic procedures to be performed. Notably, our youngest patient was a 4‐month‐old with a diagnosis of eosinophilic gastritis, with no re‐bleeding observed. HS was performed on the 4‐month‐old infant using a standard endoscope (Olympus) with an outer diameter of 8.9 mm and a working channel of 2.8 mm. This supports the use of HS in young children, with the youngest of 2 days old recorded in the prospective study on the use of HS in children.^9^

There are concerns of adverse events, which include abdominal pain attributed to visceral distension from the carbon dioxide (CO_2_) propellant and viscus perforation following use of HS, though in many cases it is difficult to discern whether the cause was HS, endoscope trauma, or friable tissue from the underlying condition.^12^

In many studies, HS is shown to be safe and effective for treating patients with bleeding from multiple causes of UGIB either alone, in combination with other therapeutic agents, or as rescue therapy.^7^, ^13^ Several studies have investigated the use of HS in the management of various aetiologies, including bleeding peptic ulcers, malignancy, anticoagulated patients and variceal bleeding with encouraging haemostasis rates (65%–98%).^14^, ^15^, ^16^, ^17^ Acute variceal bleeding was not included in our review, though the use of HS has been reported in variceal bleeding with a high haemostasis rate of 92.7% and a re‐bleeding rate of only 3.1%.^8^

The use of HS as a monotherapy has previously been discouraged. For peptic ulcer bleeding, their use is recommended as rescue after failure of conventional treatment or in patients with malignant bleeds or ulcers where conventional modalities are ineffective. Existing international guidelines recommend combination therapy (i.e., injection in addition to either thermal or mechanical haemostasis techniques) for patients with peptic ulcers with stigmata that suggest a high risk of re‐bleeding.^18^

There has been encouraging data on HS's efficacy as a single agent. For peptic ulcers, HS was shown to be non‐inferior to conventional dual therapy in a prospective study of 224 patients, with re‐bleeding rate of 12.5% in patients treated with HS as monotherapy versus 15.4% with conventional therapy.^19^ In another study, haemostasis was achieved at the index endoscopy in 90.9% of patients (60/66) with HS alone. Re‐bleeding occurred in 13.3% of patients (8/60), with higher rates of early bleeding in patients with Forrest Ia ulcers.^20^ HS may prove to be an effective initial treatment for patients with active peptic ulcer bleeding, with the new American College of Gastroenterology guidelines making a conditional recommendation for its use for actively bleeding ulcers.^21^

## 5 CONCLUSION

Further research is necessary to confirm if haemostatic powder spray can be used as monotherapy, especially in patients with actively spurting bleeding ulcers. In paediatric patients, monotherapy with HS has been shown to be as effective as conventional approaches in the management of children with UGIB with no adverse events encountered.^9^ Similarly, the findings of our audit also support the efficacy and success of HS as a monotherapy.

Limitations of our audit are the retrospective analysis, as well as the lack of a control group. Nonetheless it provides information on important real‐world application of HS in children and is an important snapshot on current treatment of UGIB in our centre.

In conclusion, the findings of our audit support the use of HS in the management of UGIB in children. HS, either as monotherapy or in combination with other conventional therapy, has the potential to improve outcomes of UGIB in children, due to its ease of use, allowing for wider and more timely access to life‐saving endoscopic therapy.

## CONFLICT OF INTEREST STATEMENT

The authors declare no conflict of interest.
